# Evaluation of participatory teaching methods in undergraduate medical students’ learning along the first academic courses

**DOI:** 10.1371/journal.pone.0190173

**Published:** 2018-01-18

**Authors:** Beatriz Gal, Margarita Rubio, Eva Iglesias, Purificación González

**Affiliations:** 1 Departamento de Ciencias Biomédicas Básicas, Facultad de Ciencias Básicas y de la Salud, Universidad Europea de Madrid, Villaviciosa de Odón, Madrid, Spain; 2 Departamento de Farmacia y Biotecnología, Facultad de Ciencias Básicas y de la Salud, Universidad Europea de Madrid, Villaviciosa de Odón, Madrid, Spain; 3 Departamento de Enfermería y Fisioterapia, Facultad de Enfermería Universidad de Alcalá, Alcalá de Henares, Madrid, Spain; Monash University, AUSTRALIA

## Abstract

The European Higher Education Area (EHEA) is an opportunity to redesign medical education. Academic training is now focused on acquiring not only knowledge, but also those competencies critical to face complex professional scenarios. Together with re-evaluating traditional teaching methods, EHEA has forced a technological shift in the way we teach. By critically assessing the impact of novel teaching methodologies, we can better define biomedical education demands. Here, we address this question on a sample of medical students instructed in basic subjects along the first two academic courses. Two hundred and one medical students participated in the study (n = 128 first year, n = 73 second year). Quantitative (conventional survey statistics) and qualitative (open coding) approaches were combined to analyze data from surveys, confidential questionnaires, semi-structured interviews and open discussion. First year medical students rated more positively the use of participatory methodologies than second year students. A major drawback is detected in the perceived workload. Active teaching methodologies show a strong reliance on their time of implementation for medical students, a key aspect to be considered in the design of integrative participatory curricula along the first academic courses.

## Introduction

The EHEA stems from three basic concepts: to strengthen exchange programs after accumulated experience from the Erasmus and Socrates experience; the idea of a knowledge-based society that brings up the necessity to strategically combine education, research and development; and, finally, the creation of a common European framework to coordinate national policies [1]. Most authors who show interest in university teaching somehow raised their concerns about how to entrain professionals who are able to face future requests in a highly competitive and global environment [2]. In this context, early in the EHEA project, teaching and pedagogical communities established a set of generic and specific competencies that graduates should acquire depending on their educational and professional profiles. Under this theoretical framework, an open discussion arouse about the convenience of adapting teaching methodologies to instruct students not only on academic knowledge, but also on professional competencies [3]. This ran in parallel to a spectacular advancement of information and communication technologies aiding in the implementation of these new methodologies [4].

Throughout this process, biomedical educational has been redefined in most European countries to face current challenges in research and clinical practice [5, 6, 7, 8 and 9]. This resulted in new roles for the student-professor tandem with major changes in teaching methodologies applied in the classrooms [10, 11] and the use of participatory methodologies represents one example. Based on clinical cases, theoretical-practical seminars, experiments, computer simulations, problem-based learning, etc., students now play a more active role in their own learning process [12]. Presumably, student-based learning increases motivation, retention and leads to a deeper understanding of content material [13].

In spite of the widespread use of these new methodologies, only few studies have systematically assessed their impact in biomedical education [14]. Only by critically evaluating their impact, we will be able to identify areas of improvements. Here we discuss results obtained from a quantitative and qualitative analysis of over 200 medical students’ perception of participatory teaching methodologies and their impact on their learning process.

## Material and methods

Ethics approval for this study was obtained from the Universidad Europea de Madrid Ethics Committee. All students who participated in the study signed an informed consent which explained the aims of the study, guaranteed anonymity and explicitly declared their consent for the results to be published.

We adopted the Hounsell approach to critically evaluate teaching [15]. This approach arises from the necessity of exploring students’ perception regarding the new methodologies in order to obtain a comprehensive analysis of their impact in teaching outcomes. Under this framework, students are essential part in data collection, being the main source of feedback. Gathering information this way leads to feedback analysis and interpretation in order to generate new actions resulting in either the implementation of required changes or consolidation of current strategies.

We analyzed data from a total of 201 medical students: n = 128 first year, n = 73 second year. A mixed method approach was used. Quantitative data was collected through Likert-type questionnaires, whereas semi-structured interviews and an open discussion group provided qualitative data.

For quantitative analysis, students filled out a questionnaire, made up of 14 close–ended questions and 2 open–ended questions. Close–ended questions values ranged from 1 to 4, with 1 being ‘Strongly disagree’ and 4 ‘Strongly agree’. Question p.7 was a generic item aimed to evaluate acquisition of transverse competencies. This item broke up in 6 sections where each competence was specified. Descriptive survey results are shown as mean and standard deviation. Student’s t-test was used for comparisons between groups. Significant values less than 0.05 were considered statistically significant. The statistical analysis of the survey results was performed using IBM SPSS Statistics for Windows, Version 20.0 software. Armonk, NY: IBM Corp. A copy of the survey questions in Spanish and English is provided (see S1 Table)

The interviews and the open discussion group were supervised by a principal professor, who was in charge of explaining procedures to the students and collecting signed informed consents. This principal professor was not involved in the program design and therefore remained unbiased towards the survey and interview results. Guided by survey results and due to our implicit design, only a subset of students was invited to participate. The rise of an implicit approach in qualitative studies has allowed researchers to gain additional insights by relying on a number of individuals who may report on a particular question emerging from the quantitative study [16]. Given the strong temporal effect (first versus second year) detected in survey results, two different groups were organized: 3 first year medical students participated in the first interview and discussion group, and 6 second year medical students participated in the second. We have made a total of 2 interviews and 2 focus group discussion, each per group. Qualitative data analysis was performed using Atlas Ti (version 7.0), and open coding [17].

## Results

### Quantitative analysis: First and second year medical students

We looked for specific differences regarding the impact of participatory methodologies in medical students along the first two academic years. A priori, first year students are less familiar with these type of methodologies than second year students, which should influence their assessment. Table 1 summarizes mean results from all medical students evaluated. All data underlying means and statistical analysis is provided in S2 Table.

**Table 1:** Average value response (mean ± standard deviation).

Questions	Medical students (n = 201)
Compared to traditional approaches, new teaching methodologies applied in some subjects have (1-strongly disagree; 4-strongly agree)	
1. An increase in my interest in this subject	2,8 ± 0.8
2. That I feel more motivated when it comes to studying these subjects	2,6 ± 0.9
3. A reduction in my learning process	2,6 ± 0.9
4. That I have become a more active student	2,9 ± 0.9
5. That I have become a more autonomous student in the process of learning	2,8 ± 0.8
6. That I learn how to consult more often and in a better way the bibliographical sources	2,5 ± 1,0
7. I think that my transversal competencies are now better with respect to:	
A. Teamwork	2,8 ± 0,9
B. Critical analysis and understanding of scientific information	2,9 ± 0.8
C. Integration of knowledge	3,0 ± 0,8
D. Synthesis of information and ability for written and oral communication	2,7 ± 0,9
E. ICT management (information and communication technologies)	2,6 ± 0,8
F. Creativity	2,4 ± 0,9
8. The knowledge acquired with these methodologies generates a better learning, than if only master classes had been taught	3,0 ± 0,1
9. The knowledge acquired with these methodologies generates a better training, than if only master classes had been given	3,0 ± 0,9
10. The new applied methodologies have resulted in a greater workload for me	2,9 ± 0,9
11. The new applied methodologies have been useful for me in more subjects	2,7 ± 0,9
12. I prefer the continuous evaluation that takes into account all the work done, instead of being evaluated only with exams	3,1 ± 1,0
13. The usual classrooms are suitable for the new active methodologies	3,2 ± 0,8
14. The number of students in my class is adequate for the new active methodologies	3,2 ± 0,9

**Fig 1:**
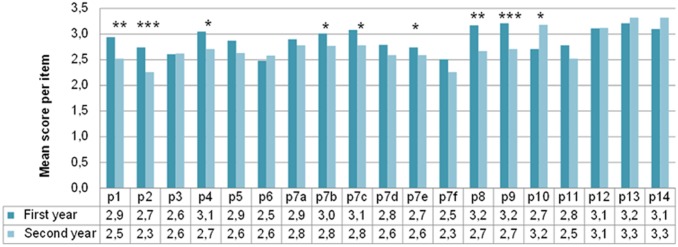
Quantitative results (mean) for each item of the survey. Data recorded from first and second years’ medical students. Asterisks indicate significant differences: * p<0.05; **p<0.001; *** p<0.0001.

Fig 1 shows survey results from first year (n = 128) and second year (n = 73) students separately. Global assessment of most items in the questionnaire was more positive for first than for second year students, finding significant differences in some critical items.

We found that active methodologies better attracted the students’ interest at first than second year course (item p1, t = 3,2; p = 0.001). They felt these methods increased their motivation (item p2; t = 3,8; p<0.001), made them better students (item p4; t = 2,7; p = 0.007) and helped them in acquiring competencies such as critical thinking and understanding (item p7b, t = 2,0; p = 0,045), better knowledge assimilation (item p7c, t = 2,5; p = 0,014) and use of communication and information technologies (item p7e, t = 2,5; p = 0,012). Finally, item p8, which investigates whether knowledge acquired with these methodologies resulted in better learning than master classes, shows a definitive degree of satisfaction of first year students (t = 3,4; p = 0,001) as well as item p9, which asked whether it resulted in better training than master classes (t = 3,7; p<0.0001). This trend for better scores in first than second year switched for items p10 and p13 reaching statistical significance in item p10 (t = -3,8; p = 0,048) for which second year medical students considered that participatory methodologies meant a higher workload.

Thus, we concluded that medical students’ appreciation of the positive impact of participatory methodology change along the first two years. This temporal effect has to be critically considered when redefining actions to enhance academic practice.

### Qualitative analysis: Assessment perception bias along courses

To assess this score shift along courses we conducted a qualitative analysis to evaluate students’ perception. We adopted a qualitative approach to gather additional information regarding their poorer assessment. To this purpose we implemented a set of personal interviews and open discussion groups with 3 first year and 6 second year medical students. Students were randomly chosen to be representative of mean results. These students were not aware of survey results.

Consistent with quantitative data described above, first year medical students declared to be more motivated by the use of participatory methodologies than second year students. This clearly reflects the better scores of first year students for item p.1 in the survey, i.e. whether active methodologies increased their interest to subjects. First year students highlighted that these methods helped them be more autonomous learners and better information searchers as they were experienced elsewhere before. The words ‘autonomous’, ‘knowledge’, ‘searcher’, ‘research, ‘motivated’, ‘comprehensive’ appeared very frequently in their speeches. In addition, they realized there was a tighter connection among the different subjects providing them with a more conciliatory learning vision. This qualitative impression was directly related to the significant difference obtained in items p.7b and p.7c between first and second year medical students. First year students verbalized that adopting a more holistic vision was highly satisfactory and they pointed that it directly impacted their performance. The following quote transcribes the opinion of one of the first year student during the interview:

“I would like to stress that active methodologies are really important because, later, at home, you can do some research, and you realize that science and Medicine are something dynamic, that varies a lot. By bringing different points of view more in focus (…) you can better assimilate knowledge and have a more comprehensive vision”

(Student 3)

In contrast, second year students referred to the strong unnecessary workload active and participatory methodologies meant for them. We specifically discussed about assessment of item p.10, where a significant difference was found regarding workload with their first year peers. The analysis of the open discussion group corroborated this conclusion. Interestingly, for second year medical students using participatory methodologies meant working by themselves instead of collaborating in order to save their time. We could deduce that such a cooperative learning had little constructive sense if it can be implemented individually and that their design should be carefully considered. This conclusion was reinforced by their opinions regarding professor’s implication and participation. Second year students valued participatory methodologies in a very different way depending on whether they were well structured or not; whether a specific outcome was expected from active methodologies or not and whether they were supervised by a professor or not. Citations transcribed below show students’ considerations regarding professors’ roles in the implementation of these methodologies. These citations make reference to students’ speeches that took place during the open discussion group.

“Because students should have … a more leading role than the one they had before, but professors cannot lose the role they had (…) I think the problem is to say ‘just read it and comment with your mates’. There is implicitly no clear indication or expected outcome from the activity… just ‘it is all in our hands’…”

(Student 2)

“It is OK for us to take part of the lessons, but professors cannot lose their professor role”

(Student 5)

“So you see yourself a little lost in a classroom where you are the professor, and the professor just stares at you, that is not how it should be (…) The positive thing would be to be assigned a topic you are interested on, so that you can study about it and after that, do something with it, explain it in public (…) I think the negative thing is to hand over all responsibility of an important topic to the students.”

(Student 5)

“Professors are responsible for setting the pace and that role cannot be taken by students or anyone else.”

(Student 2)

We concluded that there was a strong influence of the expected role of professors in the way participatory activities were experienced by medical students along academic courses. This, together with key aspects regarding how activities are designed in terms of their outcome, team-working demands and the general value given to those new methodologies in reinforcing professional skills should be critically considered.

## Discussion

Our analysis revealed that participative methodologies may exhibit strong temporal bias in first and second years of medical School, an aspect that should be carefully considered in future designs.

First year students scored better the use of participatory methodologies than second year, but they seemed to associate learning with knowledge level exclusively according to poor differences to items p.3 (learning time) and p.6 (better consulting). This suggests that their perception of learning dissociates from training on skills. Furthermore, for first year students using active methodologies made them more autonomous but no differences in item p.5 suggest second year students dissociate this emotional effect from the use of these methodologies. From a pedagogical point of view, one of the most popular indexes used to measure the level of learning reached by students is Bloom’s taxonomy [18]. Bloom’s taxonomy considers the existence of three major categories to classify educational learning goals: there is taxonomy to measure goals based on knowledge, a second taxonomy based on action-related skills and a third one based on feelings or emotional areas. Based on this, our data suggest that a more positive appreciation of first versus second year students of active methodologies does not seem to apply transversally across Bloom’s categories.

By reinforcing the first and more traditional cognitive level, participatory methodologies may represent an educational evolution to better target a major Bloom’s axis (application, analysis, synthesis and evaluation). However, students seem to associate the use of participatory methodologies with a reduction of their time to study (item p.3) and their ability to memorize (as narrated during interviews and open discussions). This concerns were critical for second year students, who rated lower these methodologies as a tool for developing competencies (items p.7) and found them poorly useful (item p.11). This correlates with their answers to the open-ended questions, where many of them assured that one of the most notable weaknesses of participatory methodologies was that they negatively impacted in their workload. Moreover, they rose serious concerns regarding how and when these activities are planned given the different academic load in second versus first year courses. Thus the way in which these methodologies are appreciated by students, including those who rated them higher, is poorly unrelated across the three major Bloom’s categories. It is important to consider this point when thinking about how to redefine their educational goals.

Most of students and teachers today have been trained strictly on a traditional model, based on master classes where professors play a leading role, a classical conception for medical students. This model as the only way of teaching has been questioned since the 90’s and, currently, teaching communities prefer to use terms such as participatory classes, group activities, open discussions, etc [14]. However, a qualitative forward leap from a superficial to a deeper knowledge requires an active commitment from students that new methodologies have failed to engage. In this sense, both our quantitative and qualitative analysis suggest that the necessary change of mentality has not been completed yet, neither for students nor faculty, who keep on supervising and evaluating knowledge on a traditional way.

It is also interesting to highlight how the qualitative approach suggested that professors’ roles was still essential for participatory methodologies and that their appreciation by students can dramatically change when teachers assume a more mentoring role. We noted that for students the extra workload in using participatory methodologies was strongly related to the idea of working by themselves without guidelines. When the use of these methodologies was supervised, students felt that the extra workload had a better impact in their understanding. This was mentioned by first and second year medical students, who highlighted the major professors’ roles as guarantors of the success of these participatory methodologies [19, 20]. The result of our work suggest active teaching methodologies represent a strong reliance on their time of implementation for medical students, a key aspect to be considered in the design of integrative participatory curricula along academic courses.

## Conclusions

In this work we carried out a quantitative and qualitative study over more than 200 medical students, with the aim to analyze the effect and appreciation of the use of participatory teaching methodologies and their impact. The assessment of participatory methodologies shows a strong dependence on the time of their implementation. These aspects have to be taken into account when designing the curricular development, in order to achieve a better structuring of teaching methods and their effect on students’ learning.

## Supporting information

S1 Table Survey questions in Spanish and English. Survey questionnaires are shown as in original language (Spanish) and translated (English).
(DOCX)

S2 Table Survey data. Raw individual scores behind means and statistics.
(XLSX)
