# Supplementary material for: Evaluation of participatory teaching methods in undergraduate medical students’ learning along the first academic courses
Source: PLoS One. 2018 Jan 18;13(1):e0190173. doi: 10.1371/journal.pone.0190173 (PMC5773079; doi:10.1371/journal.pone.0190173)
Supplement: S1 Table — Survey questionnaires are shown as in original language (Spanish) and translated (English). (DOCX) [file pone.0190173.s001.docx]

**ANNEX 2: Satisfaction survey on active methodologies.**

**Student:**

**Grade:**

**Course:**

**Number of subjects per course in which you use active methodologies:**

This survey is part of a research project UEM OTRI 2015/UEM18 funded by the Universidad Europea and approved by university Ethics Committee. The objective of the study is to evaluate the degree of involvement and satisfaction of the students of the degrees of Medicine with said active methodologies.

Participation is voluntary and anonymous. Data confidentiality is ensured.

**In comparison with the traditional methodology, the new teaching methodologies applied in some subjects have achieved (answer questions 1-14)**

Mark with a cross the answer that you consider most appropriate.

Thank you for your cooperation.

Indicate from 1 to 4, where 1 is completely disagree and 4 totally agree, your degree of agreement with the following statements:

*** To answer the first question 1** = <10%; 2 = 10-25%; 3 = 25-50%; 4 = 50-100%

* **To answer questions 15 and 16, use the back side of the survey page.**

| Questions | Score | | | |
| --- | --- | --- | --- | --- |
|  | 1 | 2 | 3 | 4 |
| 1. An increase in my interest in this subject |  |  |  |  |
| 2. That I feel more motivated when it comes to studying these subjects |  |  |  |  |
| 3. A reduction in my learning process |  |  |  |  |
| 4. That I have become a more active student |  |  |  |  |
| 5. That I have become a more autonomous student in the process of learning |  |  |  |  |
| 6. That I learn how to consult more often and in a better way the bibliographical sources |  |  |  |  |
| 7. I think that my transversal competencies are now better with respect to: |  |  |  |  |
| 1. Teamwork |  |  |  |  |
| 1. Critical analysis and understanding of scientific information |  |  |  |  |
| 1. Integration of knowledge |  |  |  |  |
| 1. Synthesis of information and ability for written and oral communication |  |  |  |  |
| 1. ICT management (information and communication technologies) |  |  |  |  |
| 1. Creativity |  |  |  |  |
| 8. The knowledge acquired with these methodologies generates a better learning, than if only master classes had been taught |  |  |  |  |
| 9. The knowledge acquired with these methodologies generates a better training, than if only master classes had been given |  |  |  |  |
| 10. The new applied methodologies have resulted in a greater workload for me |  |  |  |  |
| 11. The new applied methodologies have been useful for me in more subjects |  |  |  |  |
| 12. I prefer the continuous evaluation that takes into account all the work done, instead of being evaluated only with exams |  |  |  |  |
| 13. The usual classrooms are suitable for the new active methodologies |  |  |  |  |
| 14. The number of students in my class is adequate for the new active methodologies |  |  |  |  |

Finally, name what you think is:

*15. The three main weaknesses of active methodologies

*16. The three main strengths of active methodologies

**ANEXO 2: Encuesta de satisfacción sobre las metodologías activas. Estudiante**

**ESTUDIANTE de la Universidad:**

**Titulación**

**Curso**

**Número de asignaturas por curso en las que utilizas metodologías activas:**

Esta encuesta forma parte del proyecto de investigación UEM OTRI 2015/UEM18 financiado por la Universidad Europea y aprobado por el Comité Ético de dicha universidad. El objetivo del estudio es evaluar el grado de aplicación y satisfacción de los estudiantes de Medicina con dichas metodologías activas.

Es voluntaria y anónima. Se asegura la confidencialidad de los datos

En comparación con la metodología tradicional, las nuevas metodologías docentes aplicadas en algunas asignaturas han logrado (conteste las preguntas 1-14):

Señale con una cruz la respuesta que considere más adecuada.

Gracias por su colaboración.

Indique de 1 a 4, donde 1 es muy en desacuerdo y 4 muy conforme,  su grado de acuerdo con las siguientes afirmaciones:

***Para contestar la primera pregunta** 1=<10%; 2=10-25%; 3=25-50%; 4=50-100%

***Para contestar las preguntas 15 y 16 utilice la cara posterior de la encuesta**

| Questions | Score | | | |
| --- | --- | --- | --- | --- |
|  | 1 | 2 | 3 | 4 |
| 1. Aumentar mi interés por esas asignaturas |  |  |  |  |
| 2. Que me sienta más motivado a la hora de estudiar esas asignaturas |  |  |  |  |
| 3. Reducir mi tiempo de aprendizaje |  |  |  |  |
| 4. Que yo sea un estudiante más activo |  |  |  |  |
| 5. Que yo sea un estudiante más autónomo en el aprendizaje |  |  |  |  |
| 6. Que yo aprenda a consultar más y mejor las fuentes bibliográficas |  |  |  |  |
| 7. Creo que ahora son mejores mis competencias transversales en |  |  |  |  |
| 1. Trabajo en equipo |  |  |  |  |
| 1. Análisis crítico y comprensión de la información científica |  |  |  |  |
| 1. Integración de conocimientos |  |  |  |  |
| 1. Síntesis de la información y capacidad de expresión escrita u oral |  |  |  |  |
| 1. Manejo de las TIC (tecnologías de la información y la comunicación) |  |  |  |  |
| 1. Creatividad |  |  |  |  |
| 8. Los conocimientos adquiridos con estas metodologías generan un mejor aprendizaje, que si se hubieran impartido solo clases magistrales |  |  |  |  |
| 9. Los conocimientos adquiridos con estas metodologías generan una mejor formación, que si se hubieran impartido solo clases magistrales |  |  |  |  |
| 10. Las nuevas metodologías aplicadas han supuesto una mayor carga de trabajo para mí |  |  |  |  |
| 11. Las nuevas metodologías aplicadas me han sido útiles para más asignaturas |  |  |  |  |
| 12. Prefiero la evaluación continua que tiene en cuenta todo el trabajo realizado, en vez de ser evaluado solo con exámenes |  |  |  |  |
| 13. Las aulas de clase habituales son adecuadas para las nuevas metodologías |  |  |  |  |
| 14. El número de alumnos de mi clase es adecuado para las nuevas metodologías |  |  |  |  |

Por último, nombre cuáles son a su juicio:

*15. Las tres debilidades principales de las metodologías activas

*16. Las tres fortalezas principales de las metodologías activas
